# Quantification of diffuse myocardial fibrosis in patients with resistant hypertension undergoing renal denervation versus hypertensive controls - preliminary results

**DOI:** 10.1186/1532-429X-16-S1-O65

**Published:** 2014-01-16

**Authors:** Adelina Doltra, Jan-Hendrik Hassel, Daniel Messroghli, Bernhard Schnackenburg, Philipp Stawowy, Rolf Gebker, Christopher Schneeweis, Alexander Berger, Eckart Fleck, Sebastian Kelle

**Affiliations:** 1 Cardiology, German Heart Institute Berlin, Berlin, Germany; 2 Philips Healthcare Systems, Hamburg, Germany

## Background

Renal Denervation (RDN) is a novel therapy for patients with resistant hypertension. Its cardiac effects at follow-up are currently unknown. On the other hand, T1 mapping permits the assessment of myocardial extracellular volume (ECV), a parameter proposed to quantify diffuse myocardial fibrosis and independently associated with mortality and hard cardiovascular events. Our aim was to study the effects of RDN on ECV at 6-month follow-up.

## Methods

14 patients with resistant hypertension undergoing RD (RD group) and 4 resistant hypertensive patients not undergoing RD (control group) were prospectively included. A 1.5T cardiac MR including T1 mapping pre- and post-contrast was performed before the RD procedure and at 6-month follow-up in both groups. Blood hematocrit was determined at both time points. Images were post-processed using commercial software (Qmass, Medis Medical Solutions, the Netherlands), and whole left ventricle (LV) ECV and septal ECV at baseline and at 6-month follow-up were quantified as follows: ECV = (1-hematocrit) * λ, where λ = (1/T1 myocardium post-contrast - 1/T1 myocardium pre-contrast)/(1/T1 blood post-contrast - 1/T1 blood pre-contrast).

## Results

**Figure 1:**
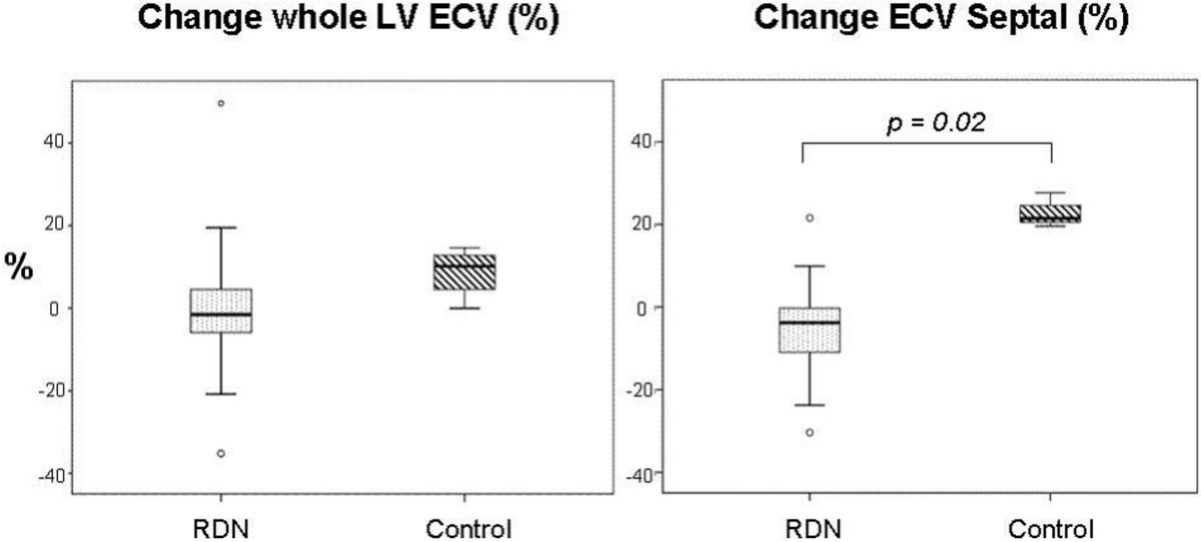
**Percent ECV-change baseline versus 6-months follow-up in patients with resistant hypertenstion undergoing renal denervation and hypertensive controls**.

No significant differences in whole LV ECV or septal ECV were observed between baseline and 6-month follow-up in the RD group. In contrast, control patients presented an increase in whole LV ECV and septal ECV at 6-month follow-up which did not reach statistical significance (p = 0.14 and p = 0.11, respectively). When the results were expressed as a % of change versus baseline, the % change of ECV septal was significantly different between the RDN and control groups (-5.4 ± 14.4 (-3.8) vs 22.9 ± 4.2 (21.5), respectively, p = 0.02; results expressed as mean ± SD (median)) (Figure 1).

## Conclusions

Extracellular space could increase at follow-up in non-RDN patients, potentially reflecting a progressive increase in myocardial fibrosis content. This effect is not observed in RDN patients, suggesting a beneficial effect of RDN in delaying this fibrotic progression. Our results are preliminary and need to be confirmed in a larger population.

## Funding

None.

